# The Association Between Knowledge, Perception, and Attitudes Towards Vitamin D and Hypovitaminosis D: A Cross-Sectional Study Conducted Among Saudi Women

**DOI:** 10.7759/cureus.75076

**Published:** 2024-12-04

**Authors:** Ambreen Hafeez, Shahd M Almatrafi, Renad I Madeni

**Affiliations:** 1 College of Medicine, United Medical and Dental College, Karachi, PAK; 2 College of Medicine, King Saud bin Abdulaziz University for Health Sciences, Jeddah, SAU

**Keywords:** attitude, fortifications, knowledge, perception, supplements, vitamin d, female

## Abstract

Background

Saudi Arabia, although it is a sunny country, has a very high rate of vitamin D deficiency, especially among females more than males. This study aimed to identify the factors associated with hypovitaminosis D among these women in order to develop effective strategies for the prevention and management of this serious health issue.

Methods

An observational cross-sectional study was conducted on 384 Saudi females in Jeddah. Participants completed an online questionnaire over six weeks, covering demographics, vitamin D knowledge, attitudes, behaviors, and perceptions of supplementation and fortified foods. The questionnaire was translated into Arabic version and then underwent a validation process using a back-translation method.

Results

The study showed that Saudi females in Jeddah had satisfactory knowledge about vitamin D, as indicated by a significantly high knowledge score. This knowledge was greater in middle-aged women (p-value 0.0369) and people with a middle income (p-value 0.0137), and it was fully independent of education level. In addition, individuals showed positive perceptions regarding vitamin D supplements and fortified foods. However, there were negative attitudes toward sun exposure, vitamin D food consumption, and getting vitamin D levels evaluated.

Conclusion

Our study found that Saudi females in Jeddah had satisfactory vitamin D knowledge, especially middle-aged women. However, there were negative attitudes toward sun exposure, vitamin D food consumption, and getting vitamin D levels evaluated. To address these concerns, healthcare professionals can play a crucial role by educating patients on the importance of vitamin D testing, dispelling myths about sun exposure, and emphasizing the benefits of vitamin D-rich diets.

## Introduction

Vitamin D plays a significant role in maintaining good health and well-being. The principal biological function of vitamin D is to promote intestinal absorption of calcium, magnesium, and phosphate for normal skeletal development and maintenance [1]. Other functions include regulation of processes such as cell development, neuromuscular and immune function, and glucose metabolism. It is also crucial for regulating several protein-encoding genes that control cell proliferation, differentiation, and apoptosis [2]. Unfortunately, there are limited food sources that contain natural vitamin D, and the best two sources are the flesh of fatty fish and fish liver oils. However, there is another way to obtain vitamin D rather than from foods, i.e., endogenously through the human skin. Exposure to ultraviolet (UV) rays from sunlight triggers vitamin D synthesis in the skin [1].

Vitamin D, which comes from diet and sunlight exposure, has two biological forms: vitamin D2 and vitamin D3. Both forms are converted by two hydroxylation reactions: one in the liver and the other in the kidney to form the active form 1, 25-dihydroxy vitamin D (calcitriol) [1]. The third way to obtain vitamin D is from dietary supplements of vitamins D2 or D. To maintain adequate vitamin D levels, healthy individuals need a daily intake of 400 IU for infants and 600 IU for adults [3]. Vitamin D deficiency and insufficiency are strongly associated with various skeletal and non-skeletal problems. Vitamin D deficiency, defined by a serum 25-hydroxyvitamin D (25(OH)D) concentration of less than 50 nmol/L (20 ng/mL), is linked to the development of rickets in children and osteomalacia and osteoporosis in adults, increasing the risk of fractures. Low vitamin D levels impair calcium and phosphate metabolism, which are crucial for bone health, leading to weakened bone structure and increased fracture susceptibility [4,5]. Besides skeletal functions, vitamin D receptors have been recognized in various tissues, indicating the roles they play in other biological functions [2]. Vitamin D deficiency has been associated with several human diseases, including hypertension, cancer, diabetes, cardiovascular and autoimmune diseases [2]. According to Bordelon et al., vitamin D deficiency affects people of all ages, although its manifestation may vary from one person to another [5]. For instance, vitamin D deficiency contributes to a range of chronic diseases, such as cardiovascular diseases, diabetes, and autoimmune disorders, while also causing musculoskeletal pain and disorders like osteomalacia and osteoporosis in others. Low vitamin D levels are associated with increased inflammation, poor immune function, and higher susceptibility to infections, further complicating overall health.

Internationally, vitamin D deficiency is highly prevalent in all countries and is considered a pandemic with a worldwide prevalence of over 1 billion [6]. Saudi Arabia, although it is a sunny country, has a very high rate of vitamin D deficiency, especially among females more than males [7,8]. Al-Daghri NM noted a systematic review that established the prevalence of vitamin D deficiency in Saudi Arabia at 81.0% [7]. According to Bokhari and Albaik, the prevalence of vitamin D deficiency among women is 78.2% for women aged 20- 50 (premenopausal) and 85% for those aged 50-79 (postmenopausal) [8]. Several factors could contribute to this high prevalence, including limited sun exposure due to cultural practices, dietary habits low in vitamin D-rich foods, insufficient supplementation, and the high prevalence of obesity, which may impair vitamin D metabolism. Additionally, age-related changes in skin synthesis and increased melanin levels in darker-skinned individuals may further exacerbate the deficiency. This study aimed to explore the knowledge, perceptions, and attitudes of Saudi women in Jeddah regarding hypovitaminosis D.

## Materials and methods

The study was an observational, cross-sectional study conducted in Jeddah, Saudi Arabia, from February to April 2024. All included participants were aged 18 years old or above, came from the general population, and different socio-economic areas. To ensure that the answers were not biased and to study the concepts of the general community, we excluded females working in the medical/paramedical field, including physicians, dentists, nurses, technicians, pharmacists, nutritionists, medical students, and any other health professionals, and females with serious illnesses such as cancer, tumors, cardiac patients who are on specific medications. From a population size of 824,910 Saudi females residing in Jeddah, a total of 384 participants took part in the study through a non-probability convenience sampling technique. The required sample size was estimated at the 95% confidence level with an estimated 50% response distribution and a margin of error of ±5%. The required minimum sample size was calculated to be 384, which accounted for a 10% nonresponse rate. Therefore, the total sample size, including the anticipated nonresponse, was considered sufficient for the study. The sample size was determined using OpenEpi's online calculator (https://www.openepi.com).

The questionnaire, adapted from O'Connor et al. (2018) [9], was translated into Arabic and then distributed to Saudi adult females using Google Forms over a period of six weeks (see Appendix). Upon opening the questionnaire, participants were asked to read and agree to the terms of an online consent form. The questionnaire was divided into four sub-sections, each containing questions related to a specific study interest. The questions were included in the form of multiple choice. 

The first sub-section asked for demographic information of the participants, including age, gender (female-male), city of residence (Jeddah or other), nationality (Saudi-non-Saudi), marital status (single- married-divorced-widowed), financial status and income, level of education, occupational information, and general health information. This section ensured confidentiality and did not ask about any names, phone numbers, or any information that exposed the participant's identity.

The second sub-section assessed the knowledge and awareness of the participants regarding vitamin D, such as what source of information they used (health professionals' books) regarding sources of vitamin D, its health benefits, and the importance and risks associated with its deficiency. 

The third sub-section evaluated the attitudes and behaviors of the participants regarding vitamin D. This section asked about the attitudes towards sun exposure: the importance of sunlight, frequency of exposure, the usage of sunscreen, skin pigmentation and its relation to vitamin D deficiency, the weather impact on getting vitamin D, and the regular behaviors when going outdoors. This section also contained questions about the nutritional habits of the participants and the vitamin D level testing.

The fourth sub-section explored the perception of vitamin D supplementation and fortified food. The participants were asked about their vitamin D supplements intake, the types of the supplements, and their concerns regarding consuming them. Also, they were asked about their perception of fortified food, its importance, and harms.

Pilot study

A preliminary assessment was conducted to evaluate the clarity, readability, and usability of the instrument before a larger study. Twenty participants completed the questionnaire twice, with a 20-minute to one-hour interval between sessions. The test-retest method assessed the reliability of the instrument by examining score stability over this short period. Internal consistency was measured using Cronbach's alpha to determine inter-item correlation. Results showed that the instrument was reliable, with a Pearson correlation coefficient of 80% and a Cronbach's alpha score exceeding 70%. Also, the questionnaire underwent content and face validity, which involved a review by three experts in the field. Based on their feedback, modifications were made to the final version of the questionnaire. The questionnaire was translated into Arabic using the back-translation method.

Ethical approval 

The Institutional Review Board (IRB) at King Abdullah International Medical Research Center (KAIMRC) approved the study with the approval number SP21J/134/03 following a detailed examination. It is important to emphasize that all women who participated in our research willingly gave their informed consent and volunteered.

Data analysis

The collected data from the completed questionnaires was entered in Microsoft Excel and checked for any typographical error before statistical analysis. The statistical analysis was performed using the JMP statistical software, version 16 (SAS Institute Inc., Cary, NC, USA). Categorical variables (such as age group and marital status) are expressed as numbers and percentages. The scoring system was as follows: 1 score was given for each right answer and 0 score was given for each wrong answer. Chi-square was used to compare the participants’ knowledge of vitamin D among demographic groups. Descriptive statistics were used to present demographic data and to evaluate knowledge, perception, and behavior regarding vitamin D. Risk factors that might be associated with vitamin D deficiency were determined using logistic regression analysis. The statistical significance of differences was considered at a p-value less than 0.05.

## Results

Characteristics of participants

Among the 587 participants who agreed to take part in the study, 203 responses were excluded from the data analysis, as they did not meet the inclusion criteria concerning gender (female), city of residence (Jeddah), nationality (Saudi), and not studying or working in the medical field. As a result, 384 replies were left for data analysis. All participants in the study were Saudi females. Table 1 presents the demographic characteristics of the samples. In terms of age, the study's major findings were that the majority of adult women were in the age groups of 18 to 28 years and 40 to 50 years (139 (36.2 %) and 118 (30.7 %), respectively), with the majority of them 231 (60.2 %) being "married”.

**Table 1:** Demographic characteristics of study participants

Demographic	Sub-Group	n= 384(%)
Age	18-28 years	139 (36.2)
	29-39 years	78 (20.3)
	40-50 years	118 (30.7)
	51-60 years	46 (12)
	>60 years	3 (0.8)
Marital Status	Single	119 (31)
	Married	231 (60.2)
	Divorced	21 (5.5)
	Widowed	13 (3.4)
Children	No children	139 (36.2)
	1	26 (6.8)
	2	36 (9.4)
	3	46 (12)
	4	69 (18)
	>4	67 (17.4)
Female-Specific Factors	Pregnant	13 (3.4)
	Menopausal	72 (18.8)
	Breastfeeding	18 (4.7)
Education Level	Did not finish high school	13 (3.4)
	High school	77 (20.1)
	Undergraduate/Bachelor’s degree	260 (67.7)
	Master’s degree	25 (6.5)
	Doctoral degree (PhD)	5 (1.3)
Income	<3000	155 (40.4)
	3000-6999	87 (22.7)
	7000-10000	69 (18)
	>10000	73 (19)
Occupation	Employed	90 (23.4)
	Unemployed	294 (76.6)
Chronic Diseases	Yes	50 (13)
	No	334 (87)

More than three-quarters of the participants had a high level of education, with 260 (67.7%) being undergraduates or having received a bachelor's degree, 25 (6.5 %) having received a master's degree, and 5 (1.3%) having received a PhD degree. On the occupational level, 294 (76.6%) of the respondents were unemployed. Furthermore, 155 (40.4 %) of respondents had an income of less than 3,000 SAR, followed by 87 (22.7 %) with a range of 3,000 to 6,999 SAR, 73 (19 %) with a salary of more than 10,000 SAR, and a comparatively close 69 (18%) with a salary of 7,000 to 10,000 SAR. In terms of chronic illness, the majority of the participants in the research 334 (87%) did not have any, compared to 50 (13%) who had either diabetes, hypertension, thyroid disorders, or cancer.

Knowledge

In terms of knowledge, all participants had heard of vitamin D. Table 2 highlights the general knowledge of vitamin D sources and daily intake, as well as the risk of deficiency among the subjects. A greater number of respondents were able to correctly choose the sources of vitamin D. The correct sources that were picked by subjects were: sunlight 376 (98%), supplements 269 (70%), and food 249 (65%). In addition, the vast majority, 318 (83%), correctly picked sunlight as the best source of vitamin D. Fewer respondents were able to identify vitamin D dietary sources. The right dietary sources were chosen as follows: oily fish 132 (34%), fortified food 120 (31%), egg yolk 105 (27%), red meat 26 (7%), and chicken 7 (2%). Only over a third of the participants, 146 (38%), were aware of the minimum recommended vitamin D intake of 10 μg/400 IU. On the other hand, many respondents were able to properly indicate who is at higher risk of developing vitamin D deficiency, with 285 (74%) choosing individuals who are not out during daylight, 89 (23%) choosing individuals in-home care, and 70 (18%) choosing individuals who cover the majority of their skin while outdoors.

**Table 2:** Knowledge of study participants regarding Vitamin D IU: International unit

	Answer	n (%)
1. Overall vitamin D sources (multiple answers):	Sunlight	376 (98)
	Water	14 (4)
	Supplements	269 (70)
	Fresh air	14 (4)
	Food	249 (65)
	Do not know	27 (6.8)
2. Best vitamin D source (one answer):	Sunlight	318 (83)
	Supplements	33 (9)
	Food	28 (7)
	Exercise	1 (0.3)
	Do not know	4 (1)
3. Vitamin D food sources (multiple answers):	Vegetables	107 (28)
	Fruits	64 (17)
	Egg yolk	105 (27)
	Red meat	26 (7)
	Oily fish	132 (34)
	Dairy products	75 (20)
	Fortified food	120 (31)
	Chicken	7 (2)
	Nuts	92 (24)
	Do not know	88 (23)
4. Vitamin D minimum recommended intake (one answer):	5μg/200 IU	132 (34)
	10μg/400 IU	146 (38)
	20μg/800 IU	71 (18)
	30μg/1600 IU	35 (9)
5. Most at risk of vitamin D deficiency (multiple answers):	Individuals who are not out during daylight.	285 (74)
	Individuals who do not eat fortified foods.	168 (44)
	Individuals who cover most of their skin while outdoors.	70 (18)
	Individuals who are in home care.	89 (23)
	Do not know	41 (11)

**Figure 1:**
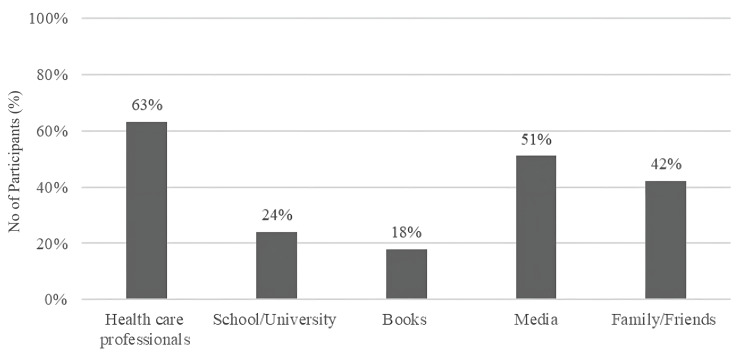
Sources of knowledge of study participants

Figure 1 illustrates the various sources of knowledge, with healthcare specialists providing the greatest information (63 %), followed by media (51%), and (42%) of people acquire their knowledge from family and friends. A small percentage receive information through school and university (24%) and books (18 %).

**Figure 2:**
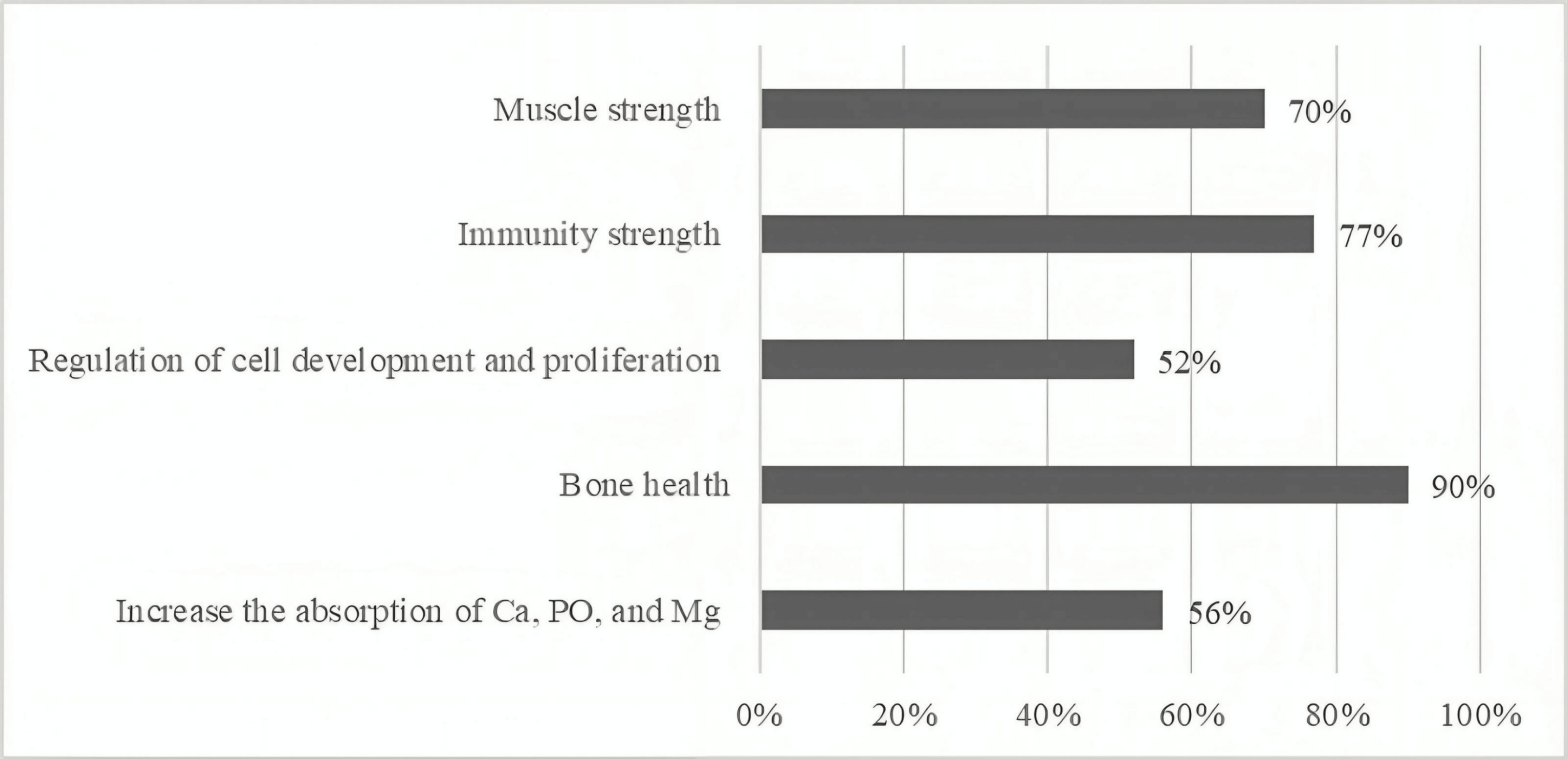
Knowledge of study participants regarding Vitamin D benefits

Figure 2 depicts the participants' understanding of the advantages of vitamin D. The outcomes were as follows: (70%) were aware that vitamin D improves muscle strength, (77%) were aware that it improves immunity, nearly half (52%) were aware that it aids in the regulation of cell development and proliferation, a vast majority (90%) were aware that it improves bone health, and (56%) were aware that it improves the absorption of Ca, Po, and Mg.

**Figure 3:**
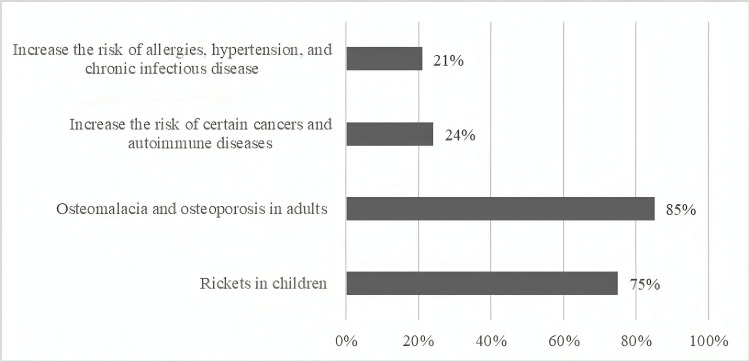
Knowledge of vitamin D deficiency problems

Figure 3 shows the subjects' knowledge of vitamin D deficiency implications. Only (21%) of those questioned believed that vitamin D deficiency increased the risk of allergies, hypertension, and chronic infectious disorders. Similarly, a portion of the respondents (24%) were aware that it raises the risk of some malignancies and autoimmune illnesses, whereas (85%) and (75%) were aware that it could cause osteoporosis and osteomalacia in adults and rickets in children, respectively.

Table 3 investigated the possible links between respondents' knowledge and demographic factors. Women aged 40-50 years had a higher satisfactory knowledge score, as seen in the table (p-value 0.0369). People with an income of 7,000 to 10,000 Saudi Riyal (SAR) had a better score as well (p-value 0.0137).

**Table 3:** Possible links between knowledge and demographics of study participants

Demographic	Knowledge		x2-test	p-value
	Satisfactory	Unsatisfactory		
Age				
18-28 years	111 (80)	28 (20)		
29-39 years	67 (86)	11 (14)	10.22	0.0351
40-50 years	106 (90)	12 (10)		
> 51	36 (73)	13 (28)		
Marital Status				
Single	97 (82)	22 (18)		
Married	197 (85)	34 (15)	2.07	0.5578
Divorced	16 (76)	5 (24)		
Widowed	10 (77)	3 (23)		
Children				
No children	111 (80)	28 (20)		
1	24 (92)	2 (8)		
2	31 (86)	5 (14)	5.12	0.4018
3	39 (85)	7 (15)		
4	61 (88)	8 (12)		
>4	53 (79)	14 (21)		
Female-Specific Factors				
Pregnant	11 (85)	2 (15)	0.01	0.8996
Menopausal	57 (79)	15 (21)	0.11	0.2926
Breastfeeding	16 (89)	2 (11)	0.42	0.5171
Education Level				
Did not finish high school	9 (69)	4 (31)		
High school	61 (79)	34 (21)		
Undergraduate/Bachelor’s degree	223 (86)	94 (14)	4.87	0.301
Master’s degree	19 (76)	9 (24)		
Doctoral degree (PhD)	4 (80)	1 (20)		
Income				
<3000	121 (78)	34 (22)		
3000-6999	72 (83)	15 (17)	10.66	0.0137
7000-10000	66 (96)	3 (4)		
>10000	61 (84)	12 (16)		
Occupation				
Employed	75 (83)	15 (17)	0	1
Unemployed	245 (83)	49 (17)		
Chronic Diseases				
Yes	43 (86)	7 (14)	0.29	0.5875
No	277 (83)	57 (17)		

Perceptions

Table 4 shows the participant's perception of vitamin D supplements and fortified food. More than half of the respondents have taken vitamin D supplements 226 (59%). The majority of the supplements taken were in the form of capsules 206 (84%). Moreover, nearly half of the participants, 132 (55%), use their supplements every week. In addition, a great number of individuals, 143 (59%), were using vitamin D supplements on the recommendation of a healthcare professional, as shown in Table 5.

**Table 4:** Perception of study participants towards Vitamin D supplements and fortified food

		Answer	n (%)
1	Take vitamin D supplements:	Yes	226 (59)
		No	158(41)
2	Type of vitamin D supplements (multiple answers):	Capsules	206 (84)
		Oil	7 (3)
		Drops	27 (11)
		Multivitamins	34 (14)
		Cod liver oil	24 (10)
		Other	0 (0)
3	Times taking vitamin D supplements:	Daily	62 (26)
		Weekly	132 (55)
		< weekly	10 (4)
		When I remember	35 (15)
		Other	0 (0)
4	Fortified food is a good source of vitamin D.	Yes	229 (60)
		No	37 (10)
		Do not know	118 (31)
5	Willing to consume fortified food to increase vitamin D levels:	Yes	232 (79)
		No	23 (8)
		Do not know	40 (14)
6	Fortified food causes health harm.	Yes	47 (12)
		No	194 (51)
		Do not know	143 (37)
7	Willing to know more about vitamin D:	Yes	290 (76)
		No	42 (11)
		Maybe	52 (14)

**Table 5:** Factors influencing the use of Vitamin D supplements

	Answer	n (%)
Reasons for taking vitamin D supplements (multiple answers):	Healthcare professional advised me to take it	143 (59)
	Do not get enough sunlight	84 (35)
	Do not eat enough vitamin D rich food	62 (26)
	To be healthy	63 (26)
	Other	0 (0)
Reasons for not taking vitamin D supplements (multiple answers):	Afraid of getting vitamin D toxicity/overdose	9 (5)
	Do not think supplements are good for my health	13 (8)
	Food is enough to get vitamin D	38 (22)
	Sun is enough to get vitamin D	21 (12)
	Too expensive	17 (10)
	Not aware of its importance	40 (24)
	Do not need it	42 (25)
	Other	0 (0)

Regarding their perception of fortified food, 229 (60%) of participants were aware that fortified food is a good source of vitamin D, and the majority, 232 (79%), were willing to consume fortified food to boost their vitamin D levels. However, 194 (51%) believed fortified food is safe, while 47 (12%) thought it was harmful, and 143 (37) were unaware of its impact on health. Almost three-quarters, 290 (76%), of the respondents wanted to learn more about vitamin D, compared to 42 (11%) who did not, as shown in Table 4.

Attitude

Table 6 describes the participant's attitudes about vitamin D insufficiency. A minority of people, 38 (10%), were able to correctly answer whether people with dark skin are more prone to vitamin D deficiency. In contrast, a vast majority, 348 (91%), were aware that sun exposure promotes the synthesis of vitamin D in the skin. 104 (27%) correctly picked that excessive sun exposure does not result in vitamin D poisoning. More than half the participants, 218 (57%), were able to correctly choose that wearing sunscreen does not lead to vitamin D deficiency. However, most responders either never 85 (22%) or rarely 138 (36%) use sunscreen.

**Table 6:** Attitudes of study participants towards vitamin D deficiency and sun exposure

Questions	Answer	n (%)
1. Dark skin is more prone to vitamin D deficiency than fair skin.	Yes	38 (10)
	No	134 (35)
	Do not know	212 (55)
2. Sun exposure promotes the production of vitamin D in the skin.	Yes	348 (91)
	No	14 (4)
	Do not know	22 (6)
3. Frequent sun exposure does not lead to vitamin D toxicity.	Yes	104 (27)
	No	95 (25)
	Do not know	185 (48)
4. Using sunscreen can lead to vitamin D deficiency.	Yes	34 (9)
	No	218 (57)
	Do not know	132 (34)
5. Sunscreen use (one answer):	Never	85 (22)
	Rarely	138 (36)
	Usually	45 (12)
	Always	48 (13)
	Sometimes	68 (18)
6. People residing in cloudy areas are more prone to vitamin D deficiency.	Yes	187 (49)
	No	49 (13)
	Do not know	148 (39)
7. Days a week spend outdoors (one answer):	1-2 days	175 (46)
	2-4 days	107 (28)
	4-6 days	57 (15)
	All week	45 (12)
8. Hours a day spent outdoors (one answer):	1-2 hours	107 (28)
	2-3 hours	62 (16)
	3-4 hours	78 (20)
	4-5 hours	54 (14)
	5-6 hours	34 (9)
	> 6 hours	49 (13)
9. Times usually stay outdoors (multiple answers):	Mornings	118 (31)
	Afternoons	83 (22)
	Evenings	263 (68)
	All day	25 (7)
10. In spending time outdoors in daylight (multiple answers):	Seek direct sun	116 (30)
	Use an umbrella	5 (1)
	Seek shadows	167 (43)
	Cover-up	164 (43)
	Other	0 (0)
11. Eating of vitamin D-rich food (one answer):	1-2 times	270 (70)
	2-4 times	96 (25)
	4-6 times	15 (4)
	> 6 times	3 (0.8)
12. Vegetarians are more likely to have vitamin D deficiency than non-vegetarians:	Yes	70 (18)
	No	70 (18)
	Do not know	244 (64)
13. Ever undergone vitamin D testing:	Yes	290 (76)
	No	42 (11)
	Planning to	52 (14)

In terms of sun exposure, almost half the participants, 187 (49%), were aware that people living in cloudy areas are more prone to vitamin D deficiency. When asked how many days they spent outdoors, the answers varied as 175 (46%) spent one to two days outdoors, 107 (28%) spent two to four days outdoors, 57 (15%) spent four to six weeks outdoors, and relatively close, 45 (12%) spent all week outdoors. Regarding the time spent outdoors, results were close as 107 (28%) spent one to two hours outside, 62 (16%) spent two to three hours, 78 (20%) spent three to four hours, 54 (14%) spent four to five hours, 34 (9%) spent five to six hours, and 49 (23%) spent > six hours outdoors. Additionally, most of the subjects, 263 (68%), tend to go outside in the evenings, but when they are outside during the day, they either seek shadows, cover-up, or seek direct sun 167 (43%), 164 (43%), and 116 (30%) respectively. In terms of eating vitamin D-rich food, the majority, 270 (70%), consume food rich in vitamin D one to two times. When asked if vegetarians are more likely to have vitamin D insufficiency, more than half of the individuals 244 (64%) did not know the answer, with the remainder evenly split between 70 (18%) properly accepting the statement and 70 (18%) disapproving the statement.

Three-quarters of subjects, 290 (76%), had their vitamin D level tested. The majority of those who took the test did so because they were concerned about its level 173 (54%) or were advised by a healthcare professional 147 (46%). In comparison, 60 (45%) of those who did not get tested did so because they were worried about having vitamin D deficiency, did not have time to be tested 40 (30%), were healthy, and did not need to 27 (20%), or did not believe it was necessary 13 (10%) (Table 7).

**Table 7:** Factors influencing Vitamin D testing behavior among study participants

	Answer	n (%)
1. Reasons for testing vitamin D (multiple answers):	Healthcare professional advised me to	147 (46)
	Friends/family advised me to	40 (12)
	Concerned about the level	173 (54)
	Other (general checkup)	2 (0.7)
2. Reasons for not testing vitamin D (multiple answers):	Concerned that I might be vitamin D deficient	60 (45)
	Do not think it is important	13 (10)
	Healthy, so I do not have to	27 (20)
	Do not have time	40 (30)
	Other	2 (2)

## Discussion

The study's findings revealed that Saudi women in Jeddah had an adequate level of general knowledge on vitamin D benefits, sources, and insufficiency, with 320 (83%) receiving satisfactory marks, which is quite excellent when compared to comparable research. For example, a study done on Bahrain, a Gulf nation with comparable geographical and racial characteristics, found that four out of every five adult Bahrainis have an insufficient understanding of vitamin D [10]. A comparable survey in Jeddah, Saudi Arabia, revealed that nearly two-thirds of the individuals had a sufficient level of understanding [11].

The findings of this study showed that all of the participants had received vitamin D information, with healthcare professionals being the most prevalent source of knowledge, which is a reliable source and validates the improved knowledge level. The majority of participants in the Bahrain research had heard about vitamin D, with the media being the most prevalent source of knowledge. This finally resulted in numerous misunderstandings and a decreased level of knowledge [10]. In the current study, a greater number of respondents were able to point out vitamin D sources, as well as identify the sun as the best source. However, there was a significant disparity in understanding of vitamin D dietary sources, with only around one-third of participants knowing that oily fish, fortified foods, and egg yolk are sources of vitamin D. Furthermore, just about 7% of those questioned were aware that red meat and chicken are sources of vitamin D as well. Moreover, 28% of individuals misidentified vegetables as a source of vitamin D, while 23 percent misidentified nuts as a source of vitamin D. Another research conducted among premenopausal women in Jeddah addressed this information gap in dietary sources similarly, stating that oily fish (48%) and fortified dairy products (36%) were properly identified as vitamin D sources by participant, only 5% of participants felt meat was a good source of vitamin D, whereas 33% said green vegetables and fruits were good ones [12].

In this research, the majority of individuals failed to indicate the recommended daily vitamin D intake, with just 38% correctly recognizing it. This might be supported by the fact that 59 % of those using vitamin D supplements were advised by their doctors to take the appropriate dosage. A very significant finding from this study is that people were able to identify people at higher risk of vitamin D deficiency, with nearly three-quarters selecting people who are not outside during daylight as people at risk, followed by 23% selecting home care patients and 18% selecting individuals who cover the majority of their skin. However, 43% of the participants stated that they seek shadows and cover-up when going outside despite their knowledge of its consequences. The great majority of people were aware that vitamin D deficiency may induce osteoporosis and osteomalacia in adults and rickets in children. In contrast, less than one-third of those who responded to the survey were aware that a lack of vitamin D raised the risk of allergies, hypertension, and chronic infectious diseases.

Similarly, just a small proportion of those questioned were aware that it increases the risk of some cancers and autoimmune diseases. In a comparable study, respondents ranked osteoporosis prevention (75.7%) and bone health (72.9%) as the most important impacts of vitamin D [12]. This study looked into the probable relationship between respondents' knowledge and demographic characteristics. Women between the ages of 40 and 50 years reported a greater level of satisfaction with their knowledge. People earning 7,000 to 10,000 SAR received a higher score as well. Surprisingly, education level was not related to knowledge level, which may be due to the fact that only 24% of individuals obtained their information from school or college, whereas the majority relied on healthcare specialists. This finding underscores the importance of incorporating nutrition education, particularly regarding vitamin D, into school and college curricula to enhance awareness and knowledge of such critical health topics.

Regarding participants' perception of vitamin D supplements, the majority of respondents were aware of vitamin D supplements, and most of them took the supplements because they were advised by healthcare professionals. These findings contraindicate a study in a Gulf Arab population that reported unfavorable perceptions toward vitamin D supplements [13]. In terms of fortified food, most of the participants had positive perceptions about food fortification, believing that it is a good source of vitamin D and willing to consume the fortified food. Similar findings were reported in a study from the UK [9].

The major findings in the participants' perceptions were that almost 91% of them believed that sun exposure boosts vitamin D production in the skin. Furthermore, nearly half of them lacked knowledge of whether excessive sun exposure causes vitamin D toxicity or not, whereas 27% accurately predicted that it did not. Similarly, barely 10% of respondents were aware that skin pigmentation is associated with a higher risk of vitamin D insufficiency. Research done in Jeddah revealed similar conclusions, with around 30.1 % agreeing that sun exposure never causes vitamin poisoning and 14.4 % stating that those with dark skin are more prone to vitamin D deficiency [11]. In the current study, more than half of the participants correctly identified that wearing sunscreen does not cause vitamin D insufficiency. However, the majority of respondents never or rarely use sunscreen. Half of the participants correctly anticipated that living in a cloudy environment increases the risk of vitamin D deficiency. However, the majority spend one to two days outside, typically in the evenings, for one to three hours. They prefer to cover themselves and seek shadows more during the day. Another research found that most persons exposed to the sun were generally in covered-up locations or shadows, giving them the mistaken impression that they were getting enough daily vitamin D supplementation [10]. In terms of dietary habits, 70% said they eat vitamin D-rich foods one to two times each day. However, when questioned about vegetarian food, 64% of respondents were unsure if vegetarians are more prone to vitamin D deficiency. Three-quarters of the respondents had their vitamin D levels checked. A key finding in this study was that worry was the primary reason for both being and not being tested, as those who had the test did so because they were concerned about their vitamin D levels. People who did not have the test, on the other hand, were anxious that they were deficient.

There are a few limitations present in this study. The use of convenience sampling restricts its ability to generalize findings to populations beyond Saudi females in Jeddah. Additionally, excluding participants from medical fields and those with severe illnesses may impact the representativeness of insights on vitamin D awareness and behaviors. Moreover, reliance on self-reported data through an online questionnaire may introduce response bias, and despite expert validation and back-translation, certain questionnaire items may still lack precision. Lastly, the cross-sectional design limits the study to identifying associations rather than establishing causation.

## Conclusions

In conclusion, the study showed that Saudi females in Jeddah had adequate knowledge about vitamin D. This knowledge was greater in middle-aged women and people with a middle income, and it was fully independent of education level. In addition, individuals showed positive perceptions regarding vitamin D supplements and fortified foods. However, there were unfavorable attitudes toward sun exposure, vitamin D food consumption, and getting vitamin D levels evaluated. Finally, we advise that concerns about vitamin D testing be addressed and managed by healthcare professionals. Providing confidence to patients and encouraging them to test their vitamin D levels for early diagnosis can help prevent the implications of vitamin D insufficiency. It is also important to consider that genetic factors may contribute to vitamin D insufficiency, influencing individual responses to supplementation and exposure to sunlight.

## Appendices

Causes of Hypovitaminosis D among Saudi Women: Knowledge, Perception or Attitude?

This study aims to identify the causes of hypovitaminosis D in Saudi women such as their knowledge regarding vitamin D (resources, health benefits, diseases associated with deficiency), perception regarding supplements and sunlight, and attitudes toward maintaining health among adult women in Jeddah, Saudi Arabia.

All the information collected from this questionnaire is confidential and will be only used for research purposes.

It takes 5-7 minutes to complete this questionnaire. Required*

**Table 8:** Vitamin D knowledge, perceptions and practices questionnaire This table presents a modified version of a previously published questionnaire on Vitamin D knowledge, perceptions, and practices. Adaptations were made to align the questionnaire with the specific objectives of this study. The original version was published under the Creative Commons Attribution (CC BY) license, permitting use and adaptation with proper attribution. Full credit is given to the original authors, O’Connor et al., 2018, and the source is cited according to the license requirements (http://creativecommons.org/licenses/by/4.0/). This adaptation has been made with acknowledgment of the terms of the CC BY license.

No.	Question Text	Options
1	Do you agree to participate in this study?	I agree
		I do not agree
2	Gender	Female
		Male
3	Age (Years)	18-28
		29-39
		40-50
		51-60
		Above 60
4	City of residence	Jeddah
		Other
5	Nationality	Saudi
		Non-Saudi
6	Marital Status	Single
		Married
		Divorced
		Windowed
7	How many children do you have?	1
		2
		3
		4
		More 4
8	Financial Status	Low income
		Moderated income
		High income
9	Level of education	Below high School
		High School
		Undergraduate/Bachelor’s degree
		Master’s degree
		Doctoral degree (PhD)
		Other
10	Are you currently working?	Yes
		No
11	If yes, are you working in the medical field?	Yes
		No
		Other
12	Are you currently pregnant, menopausal, or breastfeeding?	Yes, I am pregnant
		Yes, I am menopausal
		Yes, I am breastfeeding
		None of the above
13	Do you suffer from any serious illnesses such as cancer, tumors, diabetes, heart problems, or specific medication?	No
		Yes
14	If yes, please specify your illness or medications	[Open-ended response]
15	Have you ever heard of Vitamin D?	Yes
		No
16	If yes, where did you get your information from?	Health professionals (doctors, nurses, nutritionists)
		School/university
		Book
		Media (TV, radio, internet)
		Family/friends
		Other:
17	What are the sources of vitamin D?	Sunlight
		Water
		Supplements
		Fresh air
		Food
		Exercise
		I do not know
18	What are the best food sources of vitamin D?	Vegetables
		Fruits
		Egg yolk
		Red meat
		Oily fish
		Dairy products
		Fortified food
		Chicken
		Nuts
		I do not know
19	What is the recommended daily amount of vitamin D supplementation for adults?	5μg/200IU
		10μg/400IU
		20μg/800IU
		40μg/1600IU
20	Vitamin D is important for the absorption of calcium, phosphate, and magnesium.	Yes
		No
		I do not know
21	Vitamin D is important for the maintenance of bones and teeth.	Yes
		No
		I do not know
22	Vitamin D is important for the regulation of cell development and proliferation.	Yes
		No
		I do not know
23	Vitamin D helps to strengthen immunity.	Yes
		No
		I do not know
24	Vitamin D helps to strengthen muscles.	Yes
		No
		I do not know
25	Who are most at risk of vitamin D deficiency?	Individuals who are not out during daylight
		Individuals who do not eat fortified foods
		Individuals who cover most of their skin while outdoors
		Individuals who are in home care
		I do not know
26	Vitamin D deficiency can lead to rickets in children.	Yes
		No
		I do not know
27	Vitamin D deficiency can lead to osteomalacia and osteoporosis in adults.	Yes
		No
		I do not know
28	Vitamin D deficiency is correlated with an increased risk of certain cancers and autoimmune diseases.	Yes
		No
		I do not know
29	Vitamin D deficiency is correlated with an increased risk of allergies, hypertension, and chronic infectious diseases.	Yes
		No
		I do not know
30	Dark skin is more prone to vitamin D deficiency than fair skin.	Yes
		No
		I do not know
31	Sun exposure promotes the production of vitamin D in the skin.	Yes
		No
		I do not know
32	Frequent sun exposure does not lead to vitamin D poisoning.	Yes
		No
		I do not know
33	Using sunscreen can lead to vitamin D deficiency.	Yes
		No
		I do not know
34	How often do you wear sunscreen?	Never
		Rarely
		Usually
		Always
		Sometimes
35	People residing in cloudy areas are more prone to vitamin D deficiency.	Yes
		No
		I do not know
36	How many days a week do you spend outdoors?	1-2 days
		2-4 days
		4-6 days
		All week
37	How many hours a day do you spend outdoors?	1-2 hours
		2-3 hours
		3-4 hours
		4-5 hours
		5-6 hours
		More than 6 hours
38	At what times do you usually stay outdoors?	Mornings
		Afternoons
		Evenings
		All day
39	When spending time outdoors in daylight do you usually:	Seek direct sun
		Use an umbrella
		Seek shadows
		Cover-up
		Other:
40	How many times a week do you eat vitamin D rich food?	1-2 times
		2-4 times
		4-6 times
		More than 6 times
41	Vegetarians are more likely to have vitamin D deficiency than non-vegetarians.	Yes
		No
		I do not know
42	Have you ever had your vitamin D levels tested?	Yes
		No
		I am planning to
43	If yes, why?	Healthcare professionals advised me to
		Friends/family advised me to
		I am concerned about the level
		Other:
44	If no, why?	I am concerned that I might be vitamin D deficient
		I do not think it is important to test my vitamin D level
		I am healthy, so I do not have to
		I do not have time
		Other:
45	Do you take vitamin D supplements?	Yes
		No
46	If yes, why?	The healthcare professional advised me to take it
		I do not get enough sunlight
		I do not eat enough vitamin D-rich food
		To be healthy
		Other:
47	If yes, what type of vitamin D supplements do you use?	Capsules
		Oil
		Drops
		Multivitamins
		Cod liver oil
		Other:
48	If yes, how many times do you take vitamin D supplements?	Daily
		Weekly
		Less than weekly
		When I remember
		Other:
49	If no, why?	I am afraid of getting vitamin D toxicity/overdose
		I do not think supplements are good for my health
		I believe that food is enough to get vitamin D
		I get enough vitamin D from the sun
		Too expensive
		I am not aware of its importance
		I do not need it
		Other:
50	Do you think fortified food is a good source of vitamin D?	Yes
		No
		I do not know
51	If yes, are you willing to consume fortified food to increase your vitamin D levels?	Yes
		No
		I do not know
52	Do you think fortified food causes harm to your health?	Yes
		No
		I do not know
53	Are you willing to know more about vitamin D?	Yes
		No
		Maybe
